# Vitamin D and Abdominal Aortic Calcification in Older African American Women, the PODA Clinical Trial

**DOI:** 10.3390/nu12030861

**Published:** 2020-03-24

**Authors:** Saloni Brahmbhatt, Mageda Mikhail, Shahidul Islam, John F. Aloia

**Affiliations:** 1 Bone Mineral Research, NYU Winthrop Hospital/NYU Langone Health, Mineola, NY 11501, USA; saloni.brahmbhatt88@gmail.com; 2 Department of Endocrinology, NYU Winthrop Hospital/NYU Langone Health, Mineola, NY 11501, USA; Mageda.Mikhail@nyulangone.org; 3 Biostatistician, NYU Winthrop Research Institute, NYU Long Island School of Medicine, NYU Langone Health, Mineola, NY 11501, USA; Shahidul.Islam@nyulangone.org

**Keywords:** abdominal aortic calcification (AAC), extended aortic calcification (AAC24), chronic kidney disease (CKD), vertebral fracture assessment (VFA), vascular calcification (VC), dual-energy X-ray absorptiometry (DXA), African American (AA), parathyroid hormone

## Abstract

Abdominal aortic calcification (AAC) detected on lateral vertebral fracture assessment is associated with increased cardiovascular risk. Vitamin D deficiency and toxicity have been linked with vascular calcification. The objective of this study was to determine the effect of high-dose vitamin D on the progression of AAC. The Physical Performance, Osteoporosis and vitamin D in African American Women (PODA) is a randomized, clinical trial examining the effect of vitamin D. There were 14.7% subjects with AAC in the vitamin D group, compared to 12.1% in the placebo group at baseline. The prevalence of extended AAC at baseline was 6.4% in the vitamin D group and 3.5% in the placebo group. The extended calcification scores over time were not different between groups. There was no association between AAC and serum 25(OH)D. However, PTH was associated with an increase in AAC in the placebo group.

## 1. Introduction

Both osteoporosis and vascular calcification are common age-related findings in the elderly [1,2,3,4]. Vitamin D, interestingly, has been linked with both these processes. Vitamin D has a pivotal role not only in bone metabolism, but also in the vasculature, and may be involved in the process of vascular calcification [5,6,7]. Experts have emphasized the role of both vitamin D deficiency and excessive vitamin D in vascular toxicity, indicating a biphasic cardiovascular dose–response curve with deleterious consequences of vitamin D deficiency or excess [5,6,7,8,9,10]. Excessive vitamin D can induce vascular calcification, which can be reversed by reducing vitamin D activity [11,12,13]. It has been proposed that osteoporosis and vascular calcification have common pathophysiologic mechanisms [14].

Abdominal aortic calcification (AAC), is significantly predictive of overall cardiovascular disease incidence and mortality (e.g., coronary heart disease, stroke, congestive heart failure, and peripheral vascular disease) independently of classical risk factors [15,16,17,18,19]. AAC is also a marker of subclinical atherosclerotic disease, including coronary artery disease. It has been shown that the calcification of any artery or cardiac valve increases the risk of cardiovascular events and mortality fourfold [17,18,20,21,22,23]. Several longitudinal studies have found low plasma concentrations of 25(OH)D, associated with an increased risk of cardiovascular events [5,8,9,10]. A close relationship has also been noted between 25(OH)D and endothelial function [24,25].

Low levels of vitamin D are highly prevalent in chronic kidney disease (CKD) patients and several longitudinal studies have shown that 25(OH)D levels affect mortality independently of vascular calcification and stiffness. [23,26,27,28,29,30]. There are contrasting reports on the effect of vitamin D supplementation on vascular calcification. Some say that supplementation with Vitamin D accelerates atherosclerosis, while others support the notion that vitamin D supplementation can reverse or halt the progression of vascular calcification [11,12,31,32].

The effect of vitamin D supplementation has been studied less in healthy persons. The PODA trial had the prevention of bone loss and decline in physical performance by an intake of vitamin D sufficient to maintain 25(OH)D above 75 nmol/L as its primary aims [33,34,35]. For this report, we measured AAC every 12 months for three years to determine if high-dose vitamin D supplementation affected the progression of AAC in older African American women. We also wanted to explore the relationship between AAC and bone turnover markers and PTH. Some of these data were presented at an annual meeting of the Endocrine Society [36].

## 2. Material and Methods

The physical performance, osteoporosis prevention and vitamin D in older African Americans (PODA) study is a prospective, randomized, double-blind, placebo-controlled, three-year clinical trial of vitamin D3 supplementation in AA women older than 60 years of age [33]. Baseline demographics and laboratory values and some results have been reported in previously published papers [33,34,37,38,39,40]. In this paper, we report the results of AAC, which was a pre-specified secondary outcome. Written informed consent was obtained from each participant and the trial was approved by the Winthrop IRB and monitored by a Data Safety Monitoring Board appointed by the National Institutes of Aging. The trial was registered as NCT01153568 [41].

Healthy participants, self-declared as black, were recruited from the Long Island community. After screening, those who consented and qualified were randomly allocated to one of two groups: vitamin D3 supplementation or placebo. Inclusion criteria were serum 25(OH)D greater than 20 nmol/L and less than 65 nmol/L. Block randomization with a block size of four was performed at baseline using a computer-generated (SAS Proc Plan) randomization list. We enrolled 260 participants. Participants returned for follow-up visits every 3 months with bone density and biochemical measurements at baseline and every 6 months for 36 months. VFA images were done annually.

Participants were given either a single capsule of vitamin D or a matching placebo (depending on allocation) to take once daily. Every 3 months, the vitamin D dose was adjusted to maintain serum 25(OH)D above 75 nmol/L. The dose assignments were made in real time by the research pharmacist in consultation with the Data Coordinating Center. As doses for the active patients were titrated up or down, the blind was maintained by randomly adjusting the placebo doses to match the distribution of changes in the active patients who were at the same point in the study.

The study drug was manufactured by Alcrea Health (Pittsburgh, PA, USA). The capsules were analyzed in batches for their actual content at an independent laboratory. Calcium supplements (CaCO3) were provided, if needed, based on dietary recall, to achieve a total dietary intake of 1200 mg/day in all participants.

Serum samples were stored at −40 °C and assayed at baseline and annually for vitamin D metabolites by the Department of Laboratory Medicine at the University of Washington (Seattle, Washington) (PMID: 22968104, 21768219). Serum 25(OH)D and 1,25(OH)2D were measured by LC-MS/MS. Concentrations of 25(OH)D were standardized to NIST SRM 972a (PMID: 27091017, 22141317). The coefficient of variation (CV) of 25(OH)D measurement is 3.54%–4.41%. Serum Bone Specific Alkaline Phosphate was measured by Micro Vue BAP (Quidel Corp. San Diego, CA, USA). The kit for serum CTX was manufactured by Nordic bioscience Diagnostics A/S (Herlev, Denmark). The measurement of serum intact PTH was performed by Immulite 2000 Analyzer (Diagnostic Products Corporation, Los Angeles, CA, USA).

The primary specified endpoint for this study was a change in bone mineral density (BMD) of the total femur. Other bone density measurements included: BMD of the trochanter and femoral neck, AP spine, total body, and non-dominant radius. BMD was measured at baseline and every 6 months on a Hologic Discovery a instrument. The same instrument was used throughout the study.

### 2.1. Assessment of Aortic Calcification

All VFA scans were studied by the same reader to assess the presence of AAC. To score AAC extension, we used the score described by Kauppila et al [14,42]. The reader used the same process as described by Schousboe et al. [43]. These scores were measured at 0-, 12-, 24-, and 36-month visits. The reproducibility of the assessment was evaluated by repeated blind readings done by the same reader for all the baseline visits, and the intra-rater correlation was 0.75.

### 2.2. Statistical Analysis

Block randomization with a block size of four was performed at baseline using a computer-generated (SAS Proc Plan; SAS Institute, Inc., Cary, NC, USA) randomization list. Subjects were assigned to one of two groups: vitamin D_3_ supplementation or placebo. Any participant who was randomized and received at least one dose of the study medication was included in the intention to treat (ITT) population, and primary analysis was performed according to ITT principle. In the original study design, power was determined based on previous studies and a differential bone mineral density rate of change of 0.18% or greater per year.

Descriptive statistics were generated and presented as mean ± standard deviation or median (interquartile range) for the continuous variables, and frequency (percentage) for the categorical variables. The normality of distribution of clinical variables and laboratory markers was examined using a visual inspection of histograms and the Kolmogorov–Smirnov test. Between-group differences for each continuous variable were examined using the nonparametric Wilcoxon rank-sum test for non-normally distributed variables and the two independent samples *t*-test for normally distributed variables. Variables were checked for outliers, and analyses were performed with and without outliers, but output remained similar, so full data were used. The Fisher exact test was used to compare categorical variables between groups.

We defined an AAC 24 score of ≥5 as an extended AAC score. We also used the same as our cut-off because an AAC score of ≥5 has been shown to have a 2.4-fold increased cardiovascular disease risk compared to patients without extended AAC scores [19]. The difference in AAC rate between treatment groups over time was evaluated using a repeated-measures mixed-effects logistic regression model. Random subject specific intercept and an unstructured correlation structure was used to account for within-subject correlation between AAC rate overtime. The model included treatment groups, time and the two-way interaction effect term between time and group as the covariates. Model fit was assessed using fit statistics such as AIC and Pearson Chi-Square/DF. Associations of AAC with 25(OH)D, PTH, CTX, BAP and serum creatinine over time were also evaluated using mixed effects logistic regression models. All calculations were performed using SAS version 9.4 (SAS Institute, Inc., Cary, NC, USA). Results were considered statistically significant when *p* < 0.05.

## 3. Results

### 3.1. Baseline Demographics and Laboratory Studies 

The average age was 68.2 (65.4–72.5) years. Body mass index was similar between groups (overall 30(26.5–34.1) kg/m^2^). Few were current smokers, although 21.5% in the active group and 23.8% in the placebo group had smoked previously. In the overall sample, no statistically significant relationship was detected between serum total 25(OH)D concentration and BMI, calcium intake, BMD, muscle mass, percent body fat or measures of physical performance (SPPB balance, gait speed, chair stand score, SPPB total score, grip strength or 6MWD). Median daily calcium intake, including self-prescribed supplements, was 842 (600–1142) mg/d in the vitamin D group and 827 (628–1185) mg/d in the placebo group. Between-group PTH levels also showed no significant differences. The serum 25(OH)D values reported were obtained from LC-MS/MS. The mean dose of vitamin D3 in the active group was 3490 IU ± 1465/day. Ninety percent of the active group maintained serum 25(OH)D above 75 nmol/L.

There were no statistically significant differences in CVD risk factors between the two treatment groups: history of hypertension, hyperlipidemia, type 1 and 2 diabetes Mellitus, history of smoking and alcohol use, BMI, and use of medications such as aspirin and statins. Although the extended AAC value was higher in the vitamin D group, this was not statistically significant.

### 3.2. Efficacy of Supplementation on Abdominal Aortic Calcification (AAC)

Calcification score was dichotomized using AAC score cutoff of ≥5 [19]. There were 14.7% (16/109) subjects with the presence of AAC (calcification score > 0) in the vitamin D group compared to 12.1% (14/116) in the placebo group. The prevalence of extended AAC (AAC24 score ≥ 5) at baseline was 6.4% (7/109) in the vitamin D group and 3.5% (4/116) in the placebo group. At 36 months, the prevalence was 12.7% (9/71) in the vitamin D group and 6.9% (5/73) in the placebo group. Repeated-measures mixed effects model revealed that overall AAC did not change significantly from the baseline (slope = 0.0237, *p* = 0.091 for time effect) and was not different between treatment groups over time (non-significant interaction between group and time, slope= −0.0064, *p* = 0.791) (Table 1). Generalized chi-square/Degrees of freedom value 0.71 (value closer to one indicates better fit) for our model assures a good fit.

### 3.3. AAC, Vitamin D and Other Biomarkers

We examined the relationship between AAC and time-dependent 25(OH)D levels and other biomarkers, regardless of the treatment group assignment (Table 2). Vitamin D levels over time were not associated with AAC (*p* value = 0.220 for the time and 25(OH)D interaction). However, PTH was positively associated with calcification. As PTH increased over time, the odds of calcification (AAC ≥ 5) also increased (*p* value = 0.012 for the time and PTH interaction). Results remained comparable when adjusting the PTH model for Serum ca x Serum P product (Table 2). In addition, when the relationship of PTH with AAC over time was analyzed by group, adjusting for Serum Ca × Serum P interaction, time and PTH interactions were significant in both groups. The ‘time x serum Ca × Serum P’ product was statistically significant in the Vitamin D group (*p* = 0.046) (Table 3).

### 3.4. Incidence of Cardiovascular Disease (CVD) Risk Factors

At baseline, CVD risk factors were not significantly different between groups (Table 1). The incidence of risk factors arising during the study was as follows: 2.3% (3/130) patients developed hypertension (HTN) in each treatment group (Vitamin D and Placebo). A total of 1.5% (2/130) patients in the placebo group developed diabetes mellitus compared to none in the vitamin D group. A total of 1.5% (3/130) patients in the placebo group developed hyperlipidemia compared to 0.77% (1/130) in the vitamin D group. Combined CVD risk was calculated: 3.85% (5/130) patients developed at least one CVD risk factor in the placebo group compared to 3.08% (4/130) in the vitamin D group, with RR(95% CI) for vitamin D group compared to placebo = 0.80(0.22–2.9), *p* = 1.00.

The repeated-measures mixed-effects model revealed that overall AAC did not change significantly from the baseline (slope = 0.0232, *p* = 0.094 for time effect) and was not different between treatment groups over time (non-significant interaction between group and time, slope= −0.0059, *p* = 0.809). Incident CVD risk factors were not associated with AAC (estimate = 0.6760, *p* = 0.507). Individually, the incidence of CVD was not associated with AAC (estimate = 0.7172, *p* = 0.502). No other biomarkers, such as 1,25(OH)2D, CTX, BAP, and serum creatinine, were associated with the AAC (Table 2).

## 4. Discussion

High-dose vitamin D did not affect the progression of AAC in older African American women. In patients with chronic kidney disease and end-stage renal disease, low 25(OH)D levels are associated with vascular calcification, cardiovascular morbidity and mortality. However, very few data are available studying the relationship of vitamin D status and vascular calcification in non-chronic kidney disease pa tients. Historically, AA women have a lower prevalence of aortic and coronary calcification compared to Caucasian women, despite having higher cardiovascular events and mortality [44,45,46]. In the multi-ethnic study of atherosclerosis [44,47], the prevalence of AAC was also the highest in Caucasians (79%) compared to AAs (62%, *p* = 0.0001). In contrast, the Dallas Heart Study showed a similar prevalence of coronary calcium in AA and Caucasian men and women [48]. In the Framingham Osteoporosis study [42], the prevalence of AAC was 37% in men and 27% in women at baseline (mean age 54). At follow up after 25 years, the prevalence was 86% in both genders (mean age 79).

We used the Kauppila score for grading AAC, which is a validated, cost-effective scoring system. In a recent study, the Kauppila score has been predictive of carotid plaque (CP) and cardiac valvular calcification (CVC) with moderate accuracy in ESRD patients [49]. Additionally, there is strong evidence that AAC is predictive of CVD events and death in the general population [50]. Most studies in the past have also used CT imaging for the quantification of aortic calcification.

In our study, extended aortic calcification (AAC ≥ 5) scores were not associated with serum 25(OH)D levels or bone turnover markers such as c-terminal telopeptide, bone-specific alkaline phosphatase or serum calcium, and phosphate. However, a positive association was seen with PTH. Several clinical and experimental trials have shown a direct effect of PTH on atherogenesis via vascular remodeling and its direct actions on smooth muscle cells [51,52]. In a recent study by Pepe at al. [51], a significantly higher prevalence of AAC was noted in postmenopausal women with primary hyperparathyroidism after accounting for traditional risk factors. Another study [53] showed PTH as an independent predictor of aortic valve calcification in primary hyperparathyroidism patients after adjusting for risk factors. PTH levels are known to be higher in AA women and our study showed a positive association between PTH and extended AAC scores [54]. However, when PTH values were analyzed separately by group, it was revealed that the relationship of AAC to PTH over time was significant in the placebo group but not the vitamin D group. We previously reported that vitamin D supplementation suppressed PTH levels [35]. Vitamin D deficiency and secondary hyperparathyroidism are more prevalent in AA women compared to whites, particularly obese AA women. Higher PTH levels correlate with fat mass, fat distribution, and anthropometric measures in AA women [55,56]. The association between PTH, AAC and vitamin D needs to be explored further.

In addition, a positive association was noted between the serum Ca*P product and AAC in the vitamin D group with a trend in the placebo group. The association between an elevated Ca*P product and vascular calcification is well known, even in the absence of hyperphosphatemia [57,58,59].

Our study had several strengths and weaknesses. To the best of our knowledge, it is the first study of the effect of high-dose vitamin D supplementation on the progression of AAC in a healthy population, particularly older AA women. The study population included healthy volunteers, as we accounted for the traditional cardiovascular risk factors. This study was powered for changes in BMD (not AAC), so it is possible that a difference in AAC could have gone undetected. The prevalence of AAC and extended AAC was low for the same reason, and most likely explains the lack of any significant association seen in the present study. In comparison, a recent study of 429 Caucasian women (mean age 60 years) living in the Rabat area of Morocco had an extended AAC (AAC24 ≥ 5) prevalence of 7.9% [60].

## 5. Conclusions

There was no relationship between baseline 25(OH)D and AAC. Raising serum 25(OH)D to levels above 75 nmol/L did not influence the progression of AAC in older black women. There was an association between PTH and AAC in the placebo group, which should be explored further.

## Figures and Tables

**Table 1:** Baseline demographics and clinical characteristics.

	Vitamin D (*N* = 130)	Placebo (*N* = 130)	Overall (*N* = 260)	*p*-Value ^a^
Demographics and behavioral				
Age (years) ^b^	67.8 (65.1–71.5)	69.0 (65.4–73.4)	68.2 (65.4–72.5)	0.251
BMI (kg/m^2^) ^b^	30.2 (26.4–34.6)	30.0 (26.8–33.9)	30.1 (26.6–34.1)	0.867
Calcium intake (mg) ^b^	842.0 (600–1142)	826.5 (628.0–1185)	828.0 (614.0–1164)	0.857
Cardiovascular Risk factors				
Extended AAC (Score ≥ 5) *	7(6.4)	4(3.4)	11(4.9)	0.363
Smoking History, n (%)				0.805
Present	7(5.4)	5(3.9)	12 (4.6)	
Past	28 (21.5)	31 (23.8)	59 (22.7)	
Never	95 (73.1)	94 (72.3)	189 (72.7)	
Alcohol History, n (%)				0.323
Present	72 (55.4)	83 (63.9)	155(59.6)	
Past	2 (1.5)	2(1.5)	4(1.5)	
Never	56 (43.1)	45(34.6)	101(38.9)	
Hypertension, n (%)	87(66.9)	89(68.5)	176(67.7)	0.895
Hyperlipidemia, n (%)	44(33.9)	45(34.6)	89(34.2)	1.00
Type-I Diabetes, n (%)	1(0.77)	0(0)	1(0.38)	1.00
Type-II Diabetes, n (%)	17(13.1)	21(16.2)	38(14.6)	0.599
On Aspirin therapy, n (%)	33(25.4)	38(29.2)	71(27.3)	0.578
On Statin therapy, n (%)	37(28.5)	40(30.8)	77(29.6)	0.786
Laboratory				
Free 25OH Vitamin D (pg/mL)	4.7 ± 1.2	4.8 ± 1.3	4.7 ± 1.3	0.565
25(OH)D_3_, ng/mL	21.5 ± 6.5	22.2 ± 6.9	21.8 ± 6.7	0.352
1,25(OH)_2_D_3_, pg/mL	52.4 ± 13.7	52.6 ± 15.4	52.5 ± 14.6	0.926
PTH (pg/mL) ^b^	56.1 (41.0–73.6)	56.4 (39.5–73.8)	56.2 (39.8–73.8)	0.977
Serum Ca (mg/dL) ^b^	9.5 (9.3–9.8)	9.5 (9.3–9.8)	9.5 (9.3–9.8)	0.943
Serum Cr (mg/dL) ^b^	0.8 (0.7–0.9)	0.7 (0.6–0.9)	0.8 (0.6–0.9)	0.472
Serum P (mg/dL) ^b^	3.5 (3.2–3.8)	3.5 (3.2–3.8)	3.5 (3.2–3.8)	0.732

^a^ For continuous data, p-values are from Wilcoxon rank-sum test for non-normally distributed variables and two independent samples *t*-test for normally distributed variables. For categorical variables, p-values are from Fisher’s exact test. ^b^ Not normally distributed; IQR = Inter-quartile range (first quartile–third quartile); SD = Standard Deviation; Normally distributed variables were presented as mean ± SD and not normally distributed variables were presented as median (IQR). * abdominal aortic calcification (AAC) data available for *N* = 109 in the vitamin D group and *N* = 116 for the placebo group.

**Table 2:** Mixed effects logistic regression models for AAC (score ≥ 5) using vitamin D metabolites and other biomarkers.

Models	Estimate (Standard Error)	*p* Value
25(OH)D, ng/mL		
Time (month)	0.0466(0.0298)	0.119
25(OH)D, ng/mL	0.0463(0.0234)	0.052
Time × 25(OH)D	−0.0011(0.0009)	0.220
1,25(OH)2D, pg/mL		
Time (month)	−0.0010(0.0423)	0.982
1,25(OH)2D, pg/mL	−0.0248(0.0196)	0.205
Time × 1,25(OH)2D	0.0004(0.0008)	0.599
PTH, pg/mL		
Time (month)	−0.0468(0.0289)	0.106
PTH, pg/mL	−0.0226(0.0139)	0.105
Time × PTH	0.0014(0.0005)	0.012
PTH, pg/mL **		
Time (month)	−0.1183(0.0760)	0.120
PTH, pg/ml	−0.0236(0.0140)	0.092
Time × PTH	0.0015(0.00056)	0.007
Time × Serum Ca × Serum P	0.00193(0.0019)	0.312
CTX, ng/mL		
Time (month)	0.0098(0.0276)	0.721
CTX, ng/mL	1.0319(1.0242)	0.314
Time × CTX, ng/mL	0.0173(0.0395)	0.661
BAP, ug/mL		
Time (month)	−0.0308(0.0374)	0.411
BAP, ug/mL	−0.0563(0.0496)	0.257
Time × BAP	0.0027(0.0019)	0.165
Serum Creatinine, mg/dl		
Time (month)	0.0599(0.0763)	0.433
Serum Creatinine, mg/dL	0.4084(2.7670)	0.883
Time × Creatinine	−0.0226(0.0846)	0.790

** Secondary PTH model adjusted for ‘Serum Ca × Serum P’ product.

**Table 3:** Mixed effects logistic regression models for AAC (score ≥ 5) using PTH and product of serum C and serum P. Analysis, stratified by group.

Models	Estimate (Standard Error)	*p* Value
PTH, pg/mL (Vitamin D group)		
Time (month)	−0.2091(0.0954)	0.029
PTH, pg/mL	−0.0139(0.0158)	0.380
Time × PTH	0.0014(0.00069)	0.042
Time × Serum Ca × Serum P	0.0046(0.0023)	0.046
PTH, pg/mL (Placebo group)		
Time (month)	0.1562(0.1310)	0.234
PTH, pg/mL	−0.0492(0.0268)	0.067
Time × PTH	0.0019(0.00095)	0.041
Time × Serum Ca × Serum P	−0.0072(0.0038)	0.060

## References

[1] Hofbauer L.C., Brueck C.C., Shanahan C.M., Schoppet M., Dobnig H. (2006). Vascular calcification and osteoporosis—from clinical observation towards molecular understanding. Osteoporos. Int..

[2] Kim K.J., Kim K.M., Park K.H., Choi H.S., Rhee Y., Lee Y.-H., Cha B.-S., Kim D.Y., Oh S.M., Brown J.K. (2012). Aortic Calcification and Bone Metabolism: The Relationship between Aortic Calcification, BMD, Vertebral Fracture, 25-Hydroxyvitamin D, and Osteocalcin. Calcif. Tissue Int..

[3] Persy V., D’Haese P. (2009). Vascular calcification and bone disease: The calcification paradox. Trends Mol. Med..

[4] Vogt M.T., Valentin R.S., Forrest K.Y.-Z., Nevitt M.C., Cauley J.A. (1997). Bone Mineral Density and Aortic Calcification: The Study of Osteoporotic Fractures. J. Am. Geriatr. Soc..

[5] Nargesi A.A., Heidari B., Esteghamati S., Hafezi-Nejad N., Sheikhbahaei S., Pajouhi A., Nakhjavani M., Esteghamati A. (2016). Contribution of vitamin D deficiency to the risk of coronary heart disease in subjects with essential hypertension. Atherosclerosis.

[6] Wang J., Zhou J.J., Robertson G.R., Lee V.W. (2018). Vitamin D in Vascular Calcification: A Double-Edged Sword?. Nutrients.

[7] Zagura M., Serg M., Kampus P., Zilmer M., Eha J., Unt E., Lieberg J., Cockcroft J., Kals J. (2011). Aortic Stiffness and Vitamin D are Independent Markers of Aortic Calcification in Patients with Peripheral Arterial Disease and in Healthy Subjects. Eur. J. Vasc. Endovasc. Surg..

[8] Aggarwal R., Akhthar T., Jain S.K. (2016). Coronary artery disease and its association with Vitamin D deficiency. J. Mid-Life Health.

[9] Kunadian V., Ford G.A., Bawamia B., Qiu W., Manson J.E. (2014). Vitamin D deficiency and coronary artery disease: A review of the evidence. Am. Hear. J..

[10] Welles C.C., Whooley M.A., Karumanchi S.A., Hod T., Thadhani R., Berg A.H., Ix J.H., Mukamal K.J. (2014). Vitamin D deficiency and cardiovascular events in patients with coronary heart disease: Data from the Heart and Soul Study. Am. J. Epidemiol..

[11] Razzaque M. (2010). The dualistic role of vitamin D in vascular calcifications. Kidney Int..

[12] Wolisi G.O., Moe S.M. (2005). Vitamin D in health and disease: The Role of Vitamin D in Vascular Calcification in Chronic Kidney Disease. Semin. Dial..

[13] Zittermann A., Schleithoff S.S., Koerfer R. (2007). Vitamin D and vascular calcification. Curr. Opin. Lipidol..

[14] Kauppila L. (1997). New indices to classify location, severity and progression of calcific lesions in the abdominal aorta: A 25-year follow-up study. Atherosclerosis.

[15] Golestani R., Tio R., Zeebregts C.J., Zeilstra A., Dierckx R.A., Boersma H.H., Hillege H.L., Slart R.H.J.A. (2010). Abdominal aortic calcification detected by dual X-ray absorptiometry: A strong predictor for cardiovascular events. Ann. Med..

[16] Norman P.E., Powell J.T. (2014). Vitamin D and cardiovascular disease. Circ. Res..

[17] Okuno S., Ishimura E., Kitatani K., Fujino Y., Kohno K., Maeno Y., Maekawa K., Yamakawa T., Imanishi Y., Inaba M. (2007). Presence of Abdominal Aortic Calcification Is Significantly Associated With All-Cause and Cardiovascular Mortality in Maintenance Hemodialysis Patients. Am. J. Kidney Dis..

[18] Szulc P. (2016). Abdominal aortic calcification: A reappraisal of epidemiological and pathophysiological data. Bone.

[19] Wilson P.W.F., Kauppila L.I., O’Donnell C.J., Kiel D.P., Hannan M., Polak J.M., Cupples L.A. (2001). Abdominal Aortic Calcific Deposits Are an Important Predictor of Vascular Morbidity and Mortality. Circulation.

[20] Barreto D.V., Barreto F.C., Liabeuf S., Temmar M., Boitte F., Choukroun G., Fournier A., Massy Z.A. (2009). Vitamin D affects survival independently of vascular calcification in chronic kidney disease. Clin. J. Am. Soc. Nephrol..

[21] McCullough P.A., Sandberg K.R., Dumler F., Yanez J.E. (2004). Determinants of coronary vascular calcification in patients with chronic kidney disease and end-stage renal disease: A systematic review. J. Nephrol..

[22] Mizobuchi M., Ogata E., Koiwa F., Kinugasa E., Akizawa T. (2009). Vitamin D and vascular calcification in chronic kidney disease. Bone.

[23] Rodriguez M., Martinez-Moreno J.M., Rodríguez-Ortiz M.E., Muñoz-Castañeda J.R., Almadén Y. (2011). Vitamin D and Vascular Calcification in Chronic Kidney Disease. Kidney Blood Press. Res..

[24] Sugden J., Davies J.I., Witham M., Morris A.D., Struthers A.D. (2008). Vitamin D improves endothelial function in patients with Type 2 diabetes mellitus and low vitamin D levels. Diabet. Med..

[25] Tarcin O., Yavuz D.G., Ozben B., Telli A., Ogunc A.V., Yuksel M., Toprak A., Yazici D., Sancak S., Deyneli O. (2009). Effect of Vitamin D Deficiency and Replacement on Endothelial Function in Asymptomatic Subjects. J. Clin. Endocrinol. Metab..

[26] Levin A., Bakris G., Molitch M., Smulders M., Tian J., Williams L., Andress D. (2007). Prevalence of abnormal serum vitamin D, PTH, calcium, and phosphorus in patients with chronic kidney disease: Results of the study to evaluate early kidney disease. Kidney Int..

[27] Mehrotra R., Kermah D.A., Salusky I.B., Wolf M.S., Thadhani R.I., Chiu Y.-W., Martins D., Adler S.G., Norris K.C. (2009). Chronic kidney disease, hypovitaminosis D, and mortality in the United States. Kidney Int..

[28] Moe S.M., Chen N.X. (2007). Mechanisms of Vascular Calcification in Chronic Kidney Disease: Figure 1. J. Am. Soc. Nephrol..

[29] Schlieper G., Schurgers L., Brandenburg V., Reutelingsperger C., Floege J. (2015). Vascular calcification in chronic kidney disease: An update. Nephrol. Dial. Transpl..

[30] Vervloet M.G., Cozzolino M. (2017). Vascular calcification in chronic kidney disease: Different bricks in the wall?. Kidney Int..

[31] Hanada S., Ando R., Naito S., Kobayashi N., Wakabayashi M., Hata T., Sasaki S. (2010). Assessment and significance of abdominal aortic calcification in chronic kidney disease. Nephrol. Dial. Transpl..

[32] Honkanen E., Kauppila L., Wikström B., Rensma P.L., Krzesinski J.-M., Aasarod K., Verbeke F., Jensen P.B., Mattelaer P., Volck B. (2008). Abdominal aortic calcification in dialysis patients: Results of the CORD study. Nephrol. Dial. Transpl..

[33] Aloia J., Mikhail M., Fazzari M., Islam S., Ragolia L., Guralnik J., Ragolia L. (2018). Physical Performance and Vitamin D in Elderly Black Women–The PODA Randomized Clinical Trial. J. Clin. Endocrinol. Metab..

[34] Dhaliwal R., Mikhail M., Usera G., Stolberg A., Islam S., Ragolia L., Aloia J.F. (2018). The relationship of Physical performance and Osteoporosis prevention with vitamin D in older African Americans (PODA). Contemp. Clin. Trials.

[35] Aloia J.F., Fazzari M., Islam S., Mikhail M., Shieh A., Katumuluwa S., Dhaliwal R., Stolberg A., Usera G., Ragolia L. (2018). Vitamin D Supplementation in Elderly Black Women Does Not Prevent Bone Loss: A Randomized Controlled Trial. J. Bone Min. Res..

[36] Brahmbhatt S., Islam S., Aloia J.F. (2019). OR13-6 Hyperparathyroidism and Abdominal Aortic Calcification in Older African American Women: PODA Trial. J. Endocr. Soc..

[37] Owusu J.E., Islam S., Katumuluwa S.S., Stolberg A.R., Usera G.L., Anwarullah A.A., Shieh A., Dhaliwal R., Ragolia L., Mikhail M.B. (2018). Cognition and Vitamin D in Older African-American Women- Physical performance and Osteoporosis prevention with vitamin D in older African Americans Trial and Dementia. J. Am. Geriatr. Soc..

[38] Aloia J., Rubinova R., Fazzari M., Islam S., Mikhail M., Ragolia L. (2019). Vitamin D and Falls in Older African American Women: The PODA Randomized Clinical Trial. J. Am. Geriatr. Soc..

[39] Aloia J., Islam S., Mikhail M. (2019). Vitamin D and Acute Respiratory Infections-The PODA Trial. Open Forum Infect. Dis..

[40] Dhaliwal R., Islam S., Mikhail M., Ragolia L., Aloia J. (2020). Effect of vitamin D on bone strength in older African Americans: A randomized controlled trial. Osteoporos. Int..

[41] ClinicalTrials.gov. https://www.clinicaltrials.gov/.

[42] Kiel D.P., Kauppila L.I., Cupples L.A., Hannan M.T., O’Donnell C.J., Wilson P.W.F. (2001). Bone loss and the progression of abdominal aortic calcification over a 25 year period: The Framingham Heart Study. Calcif. Tissue Int..

[43] Schousboe J.T., Wilson K.E., Hangartner T.N. (2007). Detection of Aortic Calcification during Vertebral Fracture Assessment (VFA) Compared to Digital Radiography. PloS ONE.

[44] Farhat G.N., Cauley J.A., Matthews K., Newman A.B., Johnston J., Mackey R., Edmundowicz D., Sutton-Tyrrell K. (2006). Volumetric BMD and Vascular Calcification in Middle-Aged Women: The Study of Women’s Health Across the Nation. J. Bone Min. Res..

[45] Freedman B.I., Register T.C. (2012). Effect of race and genetics on vitamin D metabolism, bone and vascular health. Nat. Rev. Nephrol..

[46] Freedman B.I., Wagenknecht L.E., Hairston K.G., Bowden N.W., Carr J.J., Hightower R.C., Gordon E.J., Xu J., Langefeld C.D., Divers J. (2010). Vitamin D, adiposity, and calcified atherosclerotic plaque in african-americans. J. Clin. Endocrinol. Metab..

[47] Wong N.D., Lopez V.A., Allison M., Detrano R.C., Blumenthal R.S., Folsom A.R., Ouyang P., Criqui M.H. (2010). Abdominal aortic calcium and multi-site atherosclerosis: The Multiethnic Study of Atherosclerosis. Atherosclerosis.

[48] Jain T., Peshock R., McGuire D.K., Willett D., Yu Z., Vega G.L., Guerra R., Hobbs H.H., Grundy S.M. (2004). African Americans and Caucasians have a similar prevalence of coronary calcium in the Dallas Heart Study. J. Am. Coll. Cardiol..

[49] Abraham G., Shantha G.P.S., Kumar A.A., Mancha A., Koshi R., Christopher M. (2012). Is abdominal aortic calcification score a cost-effective screening tool to predict atherosclerotic carotid plaque and cardiac valvular calcification in patients with end-stage renal disease?. Indian J. Nephrol..

[50] Gonçalves F.B., Voûte M., Hoeks S.E., Chonchol M.B., Boersma E.E., Stolker R.J., Verhagen H.J.M. (2012). Calcification of the abdominal aorta as an independent predictor of cardiovascular events: A meta-analysis. Heart.

[51] Pepe J., Diacinti D., Fratini E., Nofroni I., D’Angelo A., Pilotto R., Savoriti C., Colangelo L., Raimo O., Cilli M. (2016). High prevalence of abdominal aortic calcification in patients with primary hyperparathyroidism as evaluated by Kauppila score. Eur. J. Endocrinol..

[52] Rashid G., Bernheim J., Green J., Benchetrit S. (2007). Parathyroid hormone stimulates endothelial expression of atherosclerotic parameters through protein kinase pathways. Am. J. Physiol. Physiol..

[53] Iwata S., Walker M.D., di Tullio M.R., Hyodo E., Jin Z., Liu R., Sacco R.L., Homma S., Silverberg S.J. (2012). Aortic valve calcification in mild primary hyperparathyroidism. J. Clin. Endocrinol. Metab..

[54] Perry H.M., Horowitz M., Morley J.E., Fleming S., Jensen J., Caccione P., Miller D.K., Kaiser F.E., Sundarum M. (1996). Aging and bone metabolism in African American and Caucasian women. J. Clin. Endocrinol. Metab..

[55] Valiña-Tóth A.L.B., Lai Z., Yoo W., Abou-Samra A., Gadegbeku C.A., Flack J.M. (2009). Relationship of vitamin D and parathyroid hormone with obesity and body composition in African Americans. Clin. Endocrinol..

[56] Liu J., Musani S.K., Bidulescu A., Carr J.J., Wilson J.G., Taylor H.A., Fox C.S. (2012). Fatty liver, abdominal adipose tissue and atherosclerotic calcification in African Americans: The Jackson Heart Study. Atherosclerosis.

[57] Osuka S., Razzaque M. (2012). Can features of phosphate toxicity appear in normophosphatemia?. J. Bone Min. Metab..

[58] Ganesh S.K., Stack A.G., Levin N.W., Hulbert-Shearon T., Port F.K. (2001). Association of elevated serum PO(4), Ca x PO(4) product, and parathyroid hormone with cardiac mortality risk in chronic hemodialysis patients. J. Am. Soc. Nephrol..

[59] El-Gamasy M.A., El-Shehaby W.A., Mabrouk M.M. (2019). Early predictors of cardiac dysfunction in Egyptian children with chronic kidney disease. Ann. Pediatr. Cardiol..

[60] El Maghraoui A., Hamza T., Sadni S., El Maataoui A., Majjad A., Rezqi A., Ouzzif Z., Mounach A. (2017). Vitamin D status and abdominal aortic calcification in postmenopausal women. J. Bone Min. Metab..

